# Analyses of key gene networks controlling carotenoid metabolism in Xiangfen 1 banana

**DOI:** 10.1186/s12870-021-03415-6

**Published:** 2022-01-17

**Authors:** Chen Dong, Jiuxiang Wang, Yulin Hu, Weijun Xiao, Huigang Hu, Jianghui Xie

**Affiliations:** South Subtropical Crop Research Institute, Chinese Academy of Tropical Agricultural Science/ Key Laboratory of Tropical Fruit Biology, Ministry of Agriculture/Key Laboratory of Hainan Province for Postharvest Physiology and Technology of Tropical Horticultural Products, Zhanjiang, Guangdong 524091 China

**Keywords:** Banana, Developmental period, Carotenoid compounds, Gene networks

## Abstract

**Background:**

Banana fruits are rich in various high-value metabolites and play a key role in the human diet. Of these components, carotenoids have attracted considerable attention due to their physiological role and human health care functions. However, the accumulation patterns of carotenoids and genome-wide analysis of gene expression during banana fruit development have not been comprehensively evaluated.

**Results:**

In the present study, an integrative analysis of metabolites and transcriptome profiles in banana fruit with three different development stages was performed. A total of 11 carotenoid compounds were identified, and most of these compounds showed markedly higher abundances in mature green and/or mature fruit than in young fruit. Results were linked to the high expression of carotenoid synthesis and regulatory genes in the middle and late stages of fruit development. Co-expression network analysis revealed that 79 differentially expressed transcription factor genes may be responsible for the regulation of LCYB (lycopene β-cyclase), a key enzyme catalyzing the biosynthesis of α- and β-carotene.

**Conclusions:**

Collectively, the study provided new insights into the understanding of dynamic changes in carotenoid content and gene expression level during banana fruit development.

## Background

Banana fruits play a key role in the human diet due to their desirable palatability and high nutritional value [1, 2]. Bananas are rich in various metabolites, such as soluble sugars, vitamins, carotenoids, phenolics, and minerals [3]. Of these components, carotenoids represent a large and diverse class of biological compounds and fulfill many important physiological functions [4]. However, the mechanism underlying carotenoid biosynthesis in banana remains unclear. Carotenoids in plants can produce a series of compounds named apocarotenoids under oxidative cleavage, which confers volatile compounds to the aromatic components of flowers, leaves, and fruits, as well as the well-known phytohormones, such as abscisic acid and strigolactones [5]. Carotenoids are typically tetraterpene (C40) molecules with 40 carbon atoms and multiple conjugated double bonds [6] . These bonds enable carotenoids in the selective absorption of certain wavelengths of the visible light spectrum to give bright colors, such as yellow, orange, and red, to fruits, flowers, and vegetables [7, 8]. Thus, carotenoids have been as dyes for various industrial applications due to this property. Furthermore, carotenoids can serve as precursors for the biosynthesis of vitamin A and also provide precursors to many flavor-related compounds, which confer sensory attributes to the consumers [9]. Carotenoids have been used for the food, nutraceutical, and pharmacological industries due to their various beneficial effects on human and animal health [10].

Similar to other isoprenoids, carotenoids are synthesized via successive condensations of the five-carbon molecule isopentenyl diphosphate (IPP) and its isomer dimethylallyl diphosphate (DMAPP) [11] . Plants have two distinct routes for IPP and DMAPP biosynthesis: the cytosolic mevalonic acid and the plastid methylerythritol 4-phosphate pathways [12, 13]. Geranylgeranyl pyrophosphates (GGPP) are formed by three IPP and one DMAPP in plastids. First, the colorless carotenoid phytoene is formed by the condensation of two molecules of GGPP. Then, colorless phytoene is converted into red lycopene via a series of desaturation and isomerization. Lycopene can produce a large variety of carotenoids with different physical properties via various end-group modifications, such as α-carotene, β-carotene, zeaxanthin, and lutein [7, 14]. In addition to the structural genes, some transcription factors have been reported to be involved in the synthesis of carotenoids by regulating the expression of carotenoid biosynthetic genes, such as MADS-box [15], SBP-box [16], NAC [17], AP2/ERF [18], MYB [19], HD-Zip [20], and NF-Y [21].

Integrative analysis of metabolome and transcriptome profiles has been performed because the accumulation of metabolites is preceded by coordinated increases in the transcriptional level of relevant genes. Based on the correlation, this method has been widely applied to fig [22]., asparaguses [23], peach [24], ginkgo biloba [25], kiwifruit [26], and other plants. Nevertheless, integrated investigations on carotenoid biosynthesis characteristics and regulators are relatively few. Xiangfen 1, a novel flavonoid-rich banana germplasm, was used in this study to perform the dynamic metabolites and transcriptome analyses in banana pulp at three different developmental stages and identify the accumulation patterns of carotenoids and their underlying regulation. An understanding of dynamic changes in carotenoid content and the gene expression level during fruit development is essential for the breeding of special banana subgroups with high carotenoid contents.

## Results

### Variations among carotenoid content during banana fruit flesh development

**Fig. 1:**
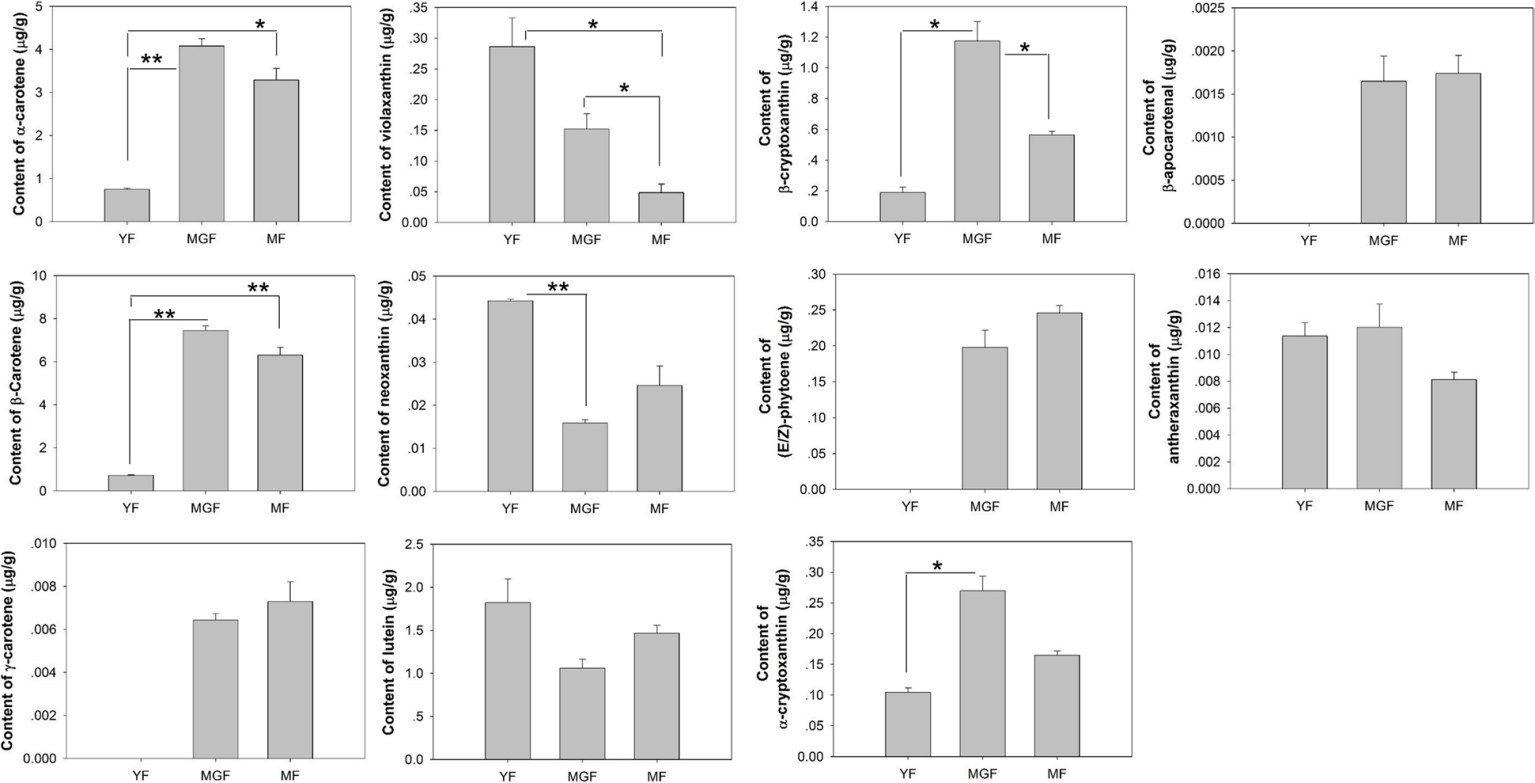
Carotenoid content (μg/g) of banana pulp across three developmental stages

As shown in Fig. 1, 11 carotenoid compounds, including α-carotene, antheraxanthin, violaxanthin, γ-carotene, neoxanthin, β-carotene, lutein, β-cryptoxanthin, β-apocarotenal, (E/Z)-phytoene, and α-cryptoxanthin, were identified from the banana pulp at different developmental stages. Most of the carotenoid compounds, such as α-carotene, β-carotene, γ-carotene, (E/Z)-phytoene, α-cryptoxanthin, β-cryptoxanthin, and β-apocarotenal were undetectable or at considerably low levels at young fruits but substantially increased at mature green and/or mature fruits (*P* < 0.05). Interestingly, the highest level of violaxanthin was observed at young fruits and then gradually decreased with fruit development (*P* < 0.05).

### Identification of differentially expressed genes (DEGs)

**Fig. 2:**
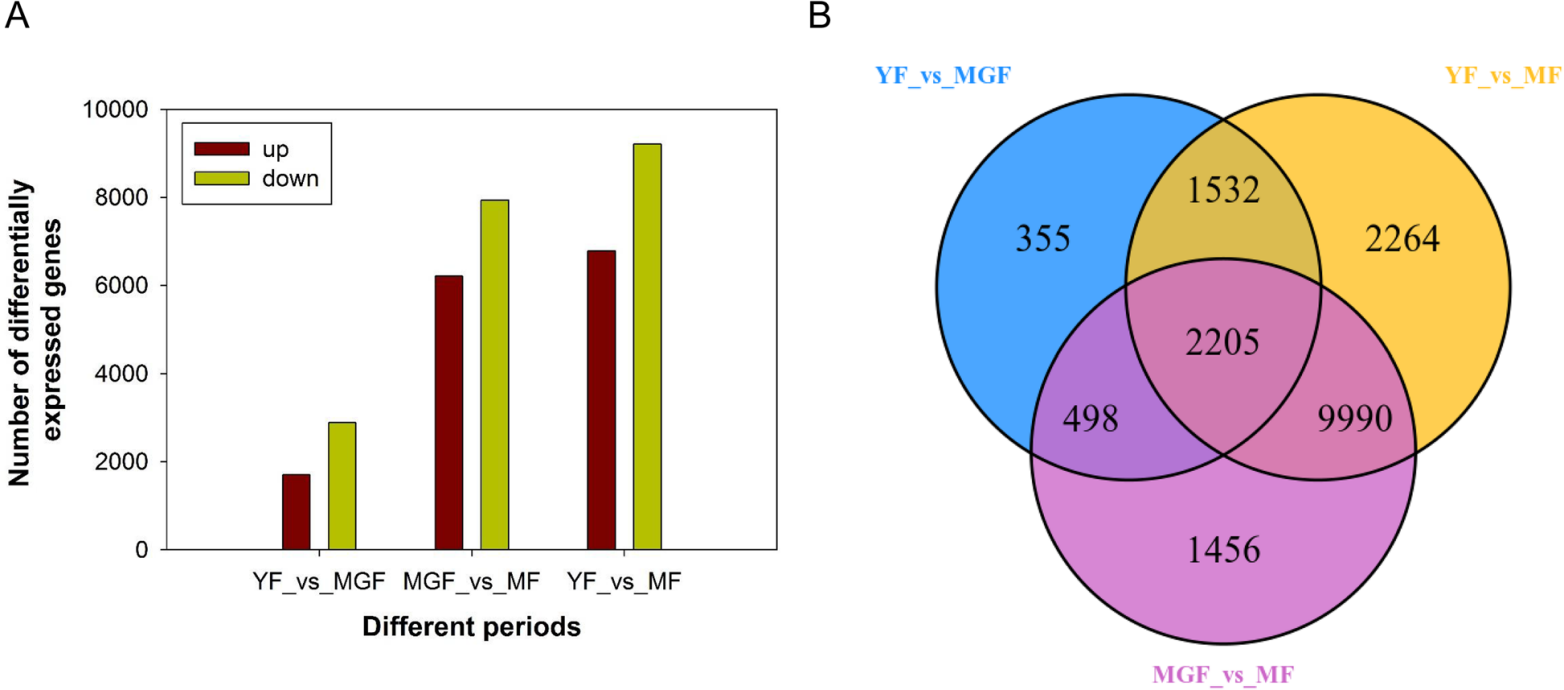
Summary of differentially expressed genes (DEGs) during fruit development. **A** Numbers of DEGs. The numbers of up-regulated genes and down-regulated genes for each comparison group are indicated with red and yellow color, respectively. **B** A Venn diagram showing the overlapping and sample-specific DEGs from the young fruit, mature green fruit, and mature fruit

Using a |log_2_ fold change| of ≥1 and an FDR of ≤0.05 as the thresholds, a total of 4590 (1703 upregulated and 2887 downregulated), 14,149 (6207 upregulated and 7942 downregulated) and 15,991 (6782 upregulated and 9209 downregulated) differentially expressed genes (DEGs) were identified in the three comparison groups: young and mature green, mature green and mature, and young and mature fruits, respectively. The majority of DEGs were downregulated during fruit development (Fig. 2A). The Venn diagram showed that 2703, 3737, and 12,195 DEGs were shared by two comparison groups, and 2205 DEGs were common to all three comparison groups (Fig. 2B).

### Enrichment of GO terms and KEGG pathway analysis

**Fig. 3:**
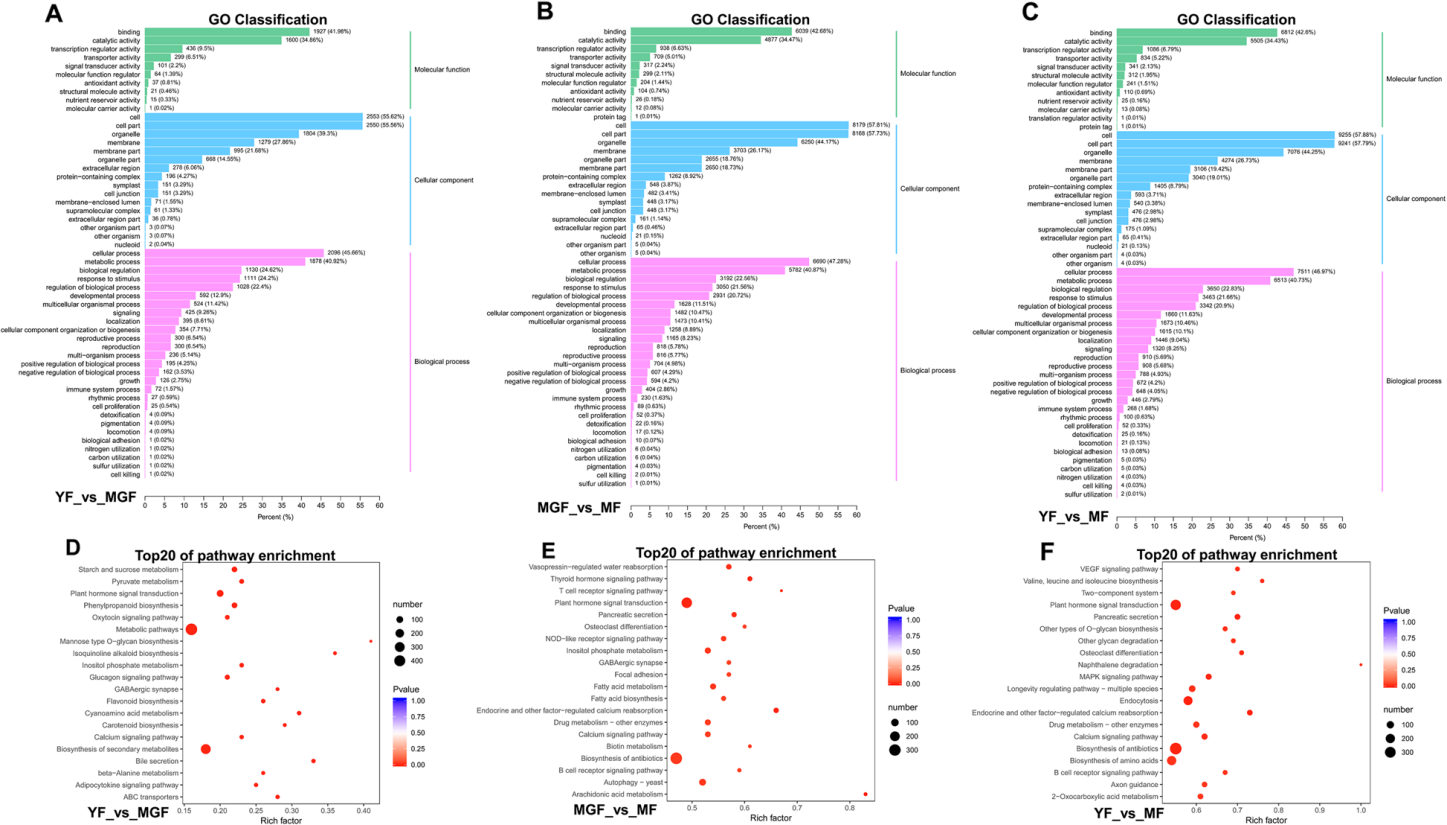
Gene-ontology (GO) classification and Kyoto Encyclopedia of Genes and Genomes (KEGG) pathway analysis of DEGs during fruit development. Functional classification of DEGs based on GO between the young fruit and mature green fruit (**A**), mature green fruit and mature fruit (**B**), and young fruit and mature fruit (**C**), respectively. KEGG pathway analysis of DEGs for the young fruit versus mature green fruit (**D**), mature green fruit versus mature fruit (**E**), and young fruit versus mature fruit (**F**)

Gene Ontology (GO) term analysis was assigned to the identified DEGs to evaluate the gene expression of fruit development (Fig. 3A, B, C). GO analysis classified 18,839, 17,800, and 17,469 genes into the biological process, cell component, and molecular function, respectively. Among the biological process categories, the cellular and metabolic processes account for a higher proportion, followed by biological regulation, response to stimulus, and regulation of biological process. The most highly represented terms within the cellular component categories were the cell, cell part, organelle, membrane, and membrane part. Meanwhile, the most highly represented terms in the molecular function categories included binding, catalytic activity, and transcription regulator activity.

The DEGs were mapped to the reference pathways in the KEGG database to obtain additional information regarding the biological pathways activated in the regulation of fruit development. Among the DEGs assigned to 309 KEGG pathways in the pairwise comparisons of the young fruit versus mature green fruit (Fig. 3D), the most highly enriched pathways included biosynthesis of secondary metabolites (ko01110, *P* = 2.6 × 10^− 12^, 297 genes), metabolic pathways (ko01100, *P* = 1.6 × 10^− 9^, 485 genes), plant hormone signal transduction (ko04075, *P* = 3.6 × 10^− 6^, 97 genes), bile secretion (ko04976, *P* = 3.5 × 10^− 5^, 20 genes), and phenylpropanoid biosynthesis (ko00940, *P* = 4.5 × 10^− 5^, 52 genes). A comparison of the DEGs between mature green and mature fruits resulted in the identification of 301 KEGG pathways (Fig. 3E). The most highly enriched pathways were arachidonic acid metabolism (ko00590, *P* = 6.18 × 10^− 5^, 20 genes), endocrine and other factor-regulated calcium reabsorption (ko04961, *P* = 2.9 × 10^− 4^, 39 genes), plant hormone signal transduction (ko04075, *P* = 2.4 × 10^− 3^, 239 genes), thyroid hormone signaling pathway (ko04919, *P* = 5.8 × 10^− 3^, 34 genes), and vasopressin-regulated water reabsorption (ko04962, *P* = 0.01, 41 genes). In the comparison of young and mature fruits (Fig. 3F), DEGs were most highly enriched in endocrine and other factor-regulated calcium reabsorption (ko04961, *P* = 1.5 × 10^− 4^, 43 genes), pancreatic secretion (ko04972, *P* = 4.1 × 10^− 4^, 45 genes), biosynthesis of antibiotics (ko01130, *P* = 6.6 × 10^− 4^, 363 genes), endocytosis (ko04144, *P* = 8 × 10^− 4^, 182 genes), and plant hormone signal transduction (ko04075, *P* = 2.7 × 10^− 3^, 267 genes). Notably, the comparison of young and mature green fruits revealed that the carotenoid biosynthesis (ko01130, *P* = 3.5 × 10^− 3^, 13 genes) was also enriched.

### Expression of genes related to carotenoid biosynthesis

**Fig. 4:**
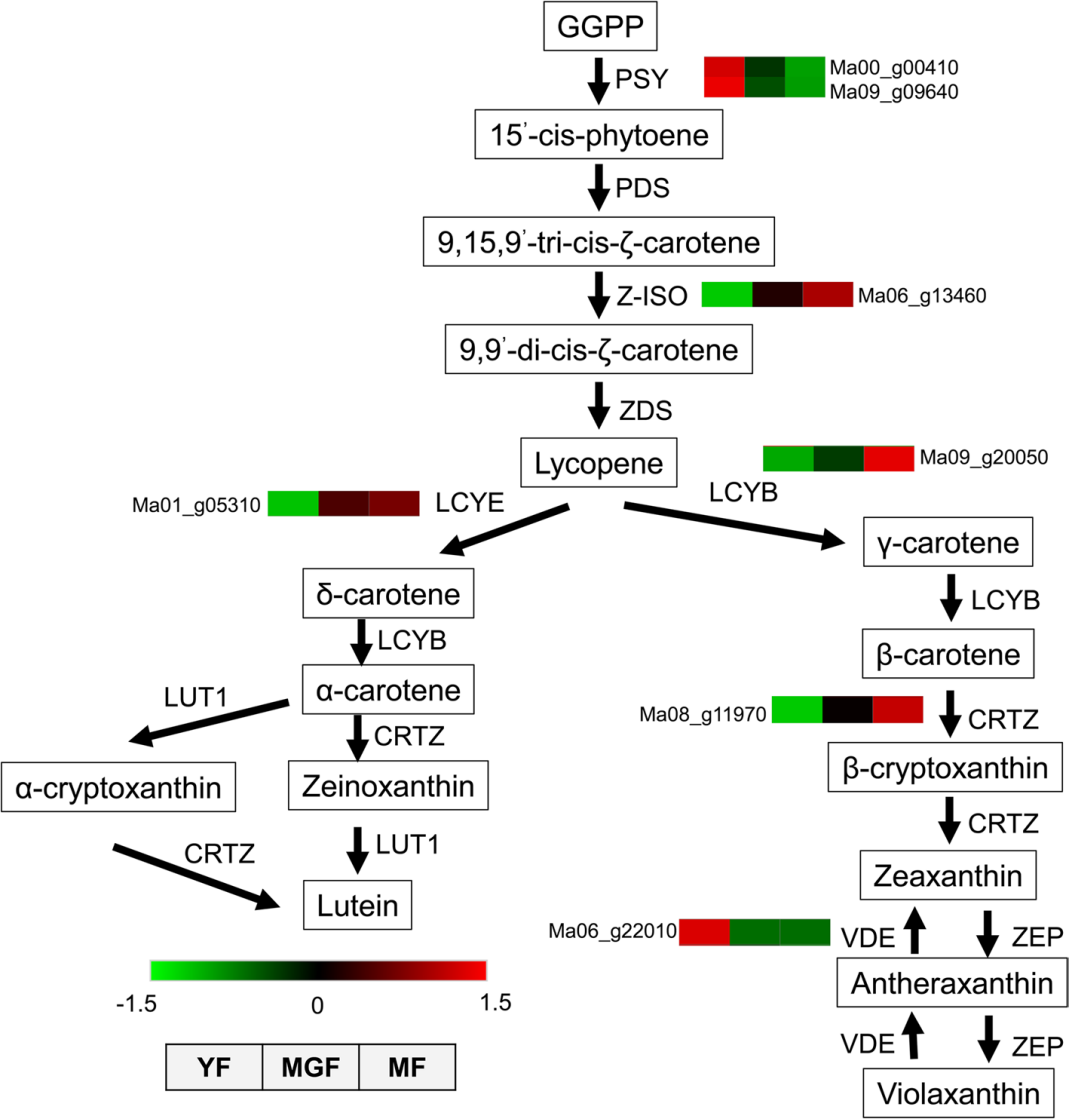
Schematic presentation of carotenoid biosynthesis. The dashed arrows represent multiple enzymatic steps. The quadrates marked with green and red background represent the reduced and increased abundances of DEGs, respectively. PSY, phytoene synthase; PDS, phytoene desaturase; Z-ISO, 15-cis-ζ-carotene isomerase; ZDS, ζ-carotene desaturase; LCYE, lycopeneε-cyclase; LCYB, lycopeneβ-cyclase; CRTZ, β-carotene 3-hydroxylase; VDE, violaxanthin de-epoxidase; ZEP, zeaxanthin epoxidase; LUT1, carotenoid epsilon hydroxylase

Carotenoid concentration is one of the main features that give an esthetic and nutritional value to banana fruit. Seven DEGs representing six genes were involved in carotenoid biosynthesis in banana in this study. The expression analysis of these DEGs is displayed in Fig. 4. The expression level of two genes encoding CRTB gradually decreased with fruit development, whereas the gene encoding Z-ISO, LCYB, LCYE, and CRTZ gradually increased during fruit development. The gene encoding VDE demonstrated high expression levels in the young fruit and low expression levels in the mature green and mature fruits.

### Transcription factors involved in carotenoid biosynthesis

Gene expression in plant carotenoid biosynthesis is strictly controlled by transcription factors. A total of 646 differentially expressed transcription factor genes were identified between the young and mature green fruits. Among these genes, 170 transcription factor genes were assigned to MADS-box (4 upregulated and 9 downregulated), SBP-box (0 upregulated and 13 downregulated), NAC (11 upregulated and 20 downregulated), AP2/ERF (16 upregulated and 29 downregulated), MYB (17 upregulated and 43 downregulated), and NF-Y (3 upregulated and 5 downregulated). Interestingly, most of the transcription factor genes demonstrated downregulation between the young and mature green fruits (Table 1).

**Table 1:** Transcription factors involved in carotenoid biosynthesis

Number	Gene ID	Family	Young fruit Expression level	Mature green fruit Expression level	regulated
1	Ma02_g02200	MADS-M-type	1096	424	down
2	Ma02_g12050	MADS-MIKC	8	165	up
3	Ma03_g08420	MADS-M-type	175	2	down
4	Ma03_g26480	MADS-MIKC	237	82	down
5	Ma03_g31640	MADS-MIKC	325	1157	up
6	Ma04_g30020	MADS-MIKC	5289	2312	down
7	Ma06_g01760	MADS-MIKC	258	92	down
8	Ma07_g00440	MADS-MIKC	122	14	down
9	Ma07_g25120	MADS-MIKC	476	75	down
10	Ma08_g04740	MADS-M-type	4	40	up
11	Ma09_g21260	MADS-MIKC	166	625	up
12	Ma11_g02670	MADS-MIKC	58	9	down
13	Ma11_g07440	MADS-MIKC	453	101	down
14	Ma02_g08090	SBP	106	49	down
15	Ma03_g10910	SBP	51	14	down
16	Ma04_g05770	SBP	143	51	down
17	Ma04_g12470	SBP	263	116	down
18	Ma05_g24390	SBP	201	86	down
19	Ma05_g25050	SBP	257	93	down
20	Ma06_g07650	SBP	10	1	down
21	Ma06_g24590	SBP	32	9	down
22	Ma08_g24570	SBP	833	189	down
23	Ma09_g16630	SBP	621	202	down
24	Ma09_g23570	SBP	52	20	down
25	Ma09_g28300	SBP	45	15	down
26	Ma11_g18010	SBP	144	52	down
27	Ma00_g01720	NAC	50	2	down
28	Ma02_g01890	NAC	595	187	down
29	Ma02_g10970	NAC	39	575	up
30	Ma03_g09370	NAC	276	1399	up
31	Ma04_g19710	NAC	1616	724	down
32	Ma05_g07350	NAC	258	49	down
33	Ma05_g07360	NAC	758	214	down
34	Ma05_g20080	NAC	18	2	down
35	Ma05_g20400	NAC	554	269	down
36	Ma05_g21000	NAC	9	0	down
37	Ma05_g29000	NAC	64	11	down
38	Ma06_g03480	NAC	9	56	up
39	Ma06_g19100	NAC	140	34	down
40	Ma06_g25140	NAC	135	620	up
41	Ma06_g27580	NAC	1189	376	down
42	Ma06_g28730	NAC	104	316	up
43	Ma06_g33980	NAC	114	6191	up
44	Ma07_g24800	NAC	5505	11,884	up
45	Ma07_g27560	NAC	3026	1430	down
46	Ma08_g09680	NAC	46	118	up
47	Ma09_g01160	NAC	797	52	down
48	Ma09_g01850	NAC	0	8	up
49	Ma09_g19410	NAC	200	14	down
50	Ma09_g24910	NAC	144	45	down
51	Ma09_g28160	NAC	136	25	down
52	Ma09_g30350	NAC	26	73	up
53	Ma11_g01240	NAC	151	39	down
54	Ma11_g16350	NAC	111	15	down
55	Ma11_g20940	NAC	0	9	up
56	Ma11_g21100	NAC	71	24	down
57	Ma11_g24060	NAC	95	25	down
58	Ma00_g00100	AP2/ERF-ERF	29	88	up
59	Ma01_g17470	AP2/ERF-AP2	52	10	down
60	Ma01_g20010	AP2/ERF-AP2	15	1	down
61	Ma02_g00070	AP2/ERF-ERF	39	87	up
62	Ma02_g09470	AP2/ERF-ERF	205	52	down
63	Ma02_g17400	AP2/ERF-ERF	309	55	down
64	Ma02_g23280	AP2/ERF-ERF	300	77	down
65	Ma03_g04220	AP2/ERF-ERF	434	210	down
66	Ma03_g04940	AP2/ERF-AP2	11	0	down
67	Ma03_g05830	AP2/ERF-ERF	6	0	down
68	Ma03_g08090	AP2/ERF-ERF	146	54	down
69	Ma03_g12670	AP2/ERF-ERF	593	2087	up
70	Ma03_g19980	AP2/ERF-ERF	17	2	down
71	Ma03_g23580	AP2/ERF-ERF	389	48	down
72	Ma04_g06130	AP2/ERF-AP2	96	4	down
73	Ma04_g09020	AP2/ERF-ERF	2	60	up
74	Ma04_g09890	AP2/ERF-ERF	43	3	down
75	Ma04_g17170	AP2/ERF-ERF	735	319	down
76	Ma04_g20370	AP2/ERF-ERF	139	5	down
77	Ma04_g21170	AP2/ERF-ERF	48	629	up
78	Ma04_g26920	AP2/ERF-ERF	998	2708	up
79	Ma05_g04410	AP2/ERF-AP2	239	1289	up
80	Ma05_g04880	AP2/ERF-ERF	21	110	up
81	Ma05_g26400	AP2/ERF-ERF	27	1	down
82	Ma05_g31650	AP2/ERF-AP2	268	108	down
83	Ma06_g01950	AP2/ERF-ERF	411	934	up
84	Ma06_g09740	AP2/ERF-ERF	496	1590	up
85	Ma06_g15710	AP2/ERF-ERF	47	12	down
86	Ma06_g24790	AP2/ERF-ERF	206	636	up
87	Ma06_g36350	AP2/ERF-AP2	86	242	up
88	Ma08_g01560	AP2/ERF-ERF	21	75	up
89	Ma08_g01810	AP2/ERF-AP2	14	1	down
90	Ma08_g09060	AP2/ERF-AP2	26	3	down
91	Ma08_g21180	AP2/ERF-ERF	52	1	down
92	Ma09_g03040	AP2/ERF-AP2	427	1114	up
93	Ma09_g12570	AP2/ERF-ERF	698	207	down
94	Ma10_g01280	AP2/ERF-AP2	51	17	down
95	Ma10_g01420	AP2/ERF-ERF	50	204	up
96	Ma10_g14680	AP2/ERF-ERF	13	0	down
97	Ma10_g19030	AP2/ERF-ERF	60	11	down
98	Ma10_g19470	AP2/ERF-ERF	604	2022	up
99	Ma10_g21410	AP2/ERF-ERF	49	7	down
100	Ma10_g26420	AP2/ERF-ERF	19	3	down
101	Ma10_g31080	AP2/ERF-ERF	12	1	down
102	Ma11_g20400	AP2/ERF-ERF	815	63	down
103	Ma00_g01590	MYB	457	1798	up
104	Ma01_g02530	MYB-related	173	57	down
105	Ma01_g14370	MYB	1367	404	down
106	Ma01_g17260	MYB	49	21	down
107	Ma01_g17870	MYB-related	117	284	up
108	Ma01_g19610	MYB	280	14	down
109	Ma02_g01300	MYB-related	154	20	down
110	Ma02_g05880	MYB	145	50	down
111	Ma02_g09720	MYB	19	0	down
112	Ma02_g09870	MYB	29	1	down
113	Ma02_g10870	MYB-related	8	0	down
114	Ma02_g17950	MYB	47	10	down
115	Ma02_g19770	MYB	5	27	up
116	Ma03_g07840	MYB	18	0	down
117	Ma03_g12720	MYB	344	1201	up
118	Ma03_g25780	MYB	47	11	down
119	Ma04_g12940	MYB	138	47	down
120	Ma04_g24670	MYB	0	6	up
121	Ma04_g26220	MYB	37	4	down
122	Ma05_g07450	MYB	46	1	down
123	Ma05_g08940	MYB-related	8	0	down
124	Ma05_g12030	MYB	86	7	down
125	Ma05_g23640	MYB	33	0	down
126	Ma05_g30120	MYB	107	19	down
127	Ma06_g04270	MYB	106	42	down
128	Ma06_g08910	MYB	198	88	down
129	Ma06_g11140	MYB	3	27	up
130	Ma06_g11270	MYB	39	9	down
131	Ma06_g12110	MYB	142	62	down
132	Ma06_g12160	MYB	45	102	up
133	Ma06_g16920	MYB	170	51	down
134	Ma06_g20700	MYB-related	5	20	up
135	Ma06_g33920	MYB	1	15	up
136	Ma07_g05780	MYB	87	272	up
137	Ma07_g19720	MYB	134	49	down
138	Ma07_g19880	MYB	58	24	down
139	Ma07_g23180	MYB	33	5	down
140	Ma07_g23230	MYB	840	3573	up
141	Ma07_g27070	MYB-related	2	13	up
142	Ma08_g01760	MYB	53	438	up
143	Ma08_g02180	MYB	526	1196	up
144	Ma08_g14720	MYB	37	11	down
145	Ma08_g15820	MYB	47	10	down
146	Ma08_g23390	MYB	248	1	down
147	Ma08_g25960	MYB	173	63	down
148	Ma09_g04930	MYB	62	2	down
149	Ma09_g05760	MYB-related	199	41	down
150	Ma09_g20610	MYB	537	239	down
151	Ma09_g25590	MYB	8	0	down
152	Ma09_g28270	MYB-related	239	608	up
153	Ma09_g30920	MYB-related	363	164	down
154	Ma10_g06750	MYB-related	49	165	up
155	Ma10_g14950	MYB	12	1	down
156	Ma10_g16050	MYB	125	22	down
157	Ma10_g26660	MYB	35	1	down
158	Ma11_g01360	MYB-related	77	17	down
159	Ma11_g03860	MYB	18	1	down
160	Ma11_g10680	MYB	725	12	down
161	Ma11_g14670	MYB	43	194	up
162	Ma11_g16430	MYB	91	23	down
163	Ma03_g11720	NF-YC	35	75	up
164	Ma04_g34950	NF-YA	127	45	down
165	Ma04_g38010	NF-YA	49	12	down
166	Ma07_g01080	NF-YA	474	149	down
167	Ma07_g13230	NF-YC	39	89	up
168	Ma08_g18750	NF-YA	29	8	down
169	Ma08_g22650	NF-YB	0	9	up
170	Ma11_g23990	NF-YC	43	8	down

### Co-expression network analysis of metabolites, genes, and transcription factors related to carotenoid biosynthesis

**Fig. 5:**
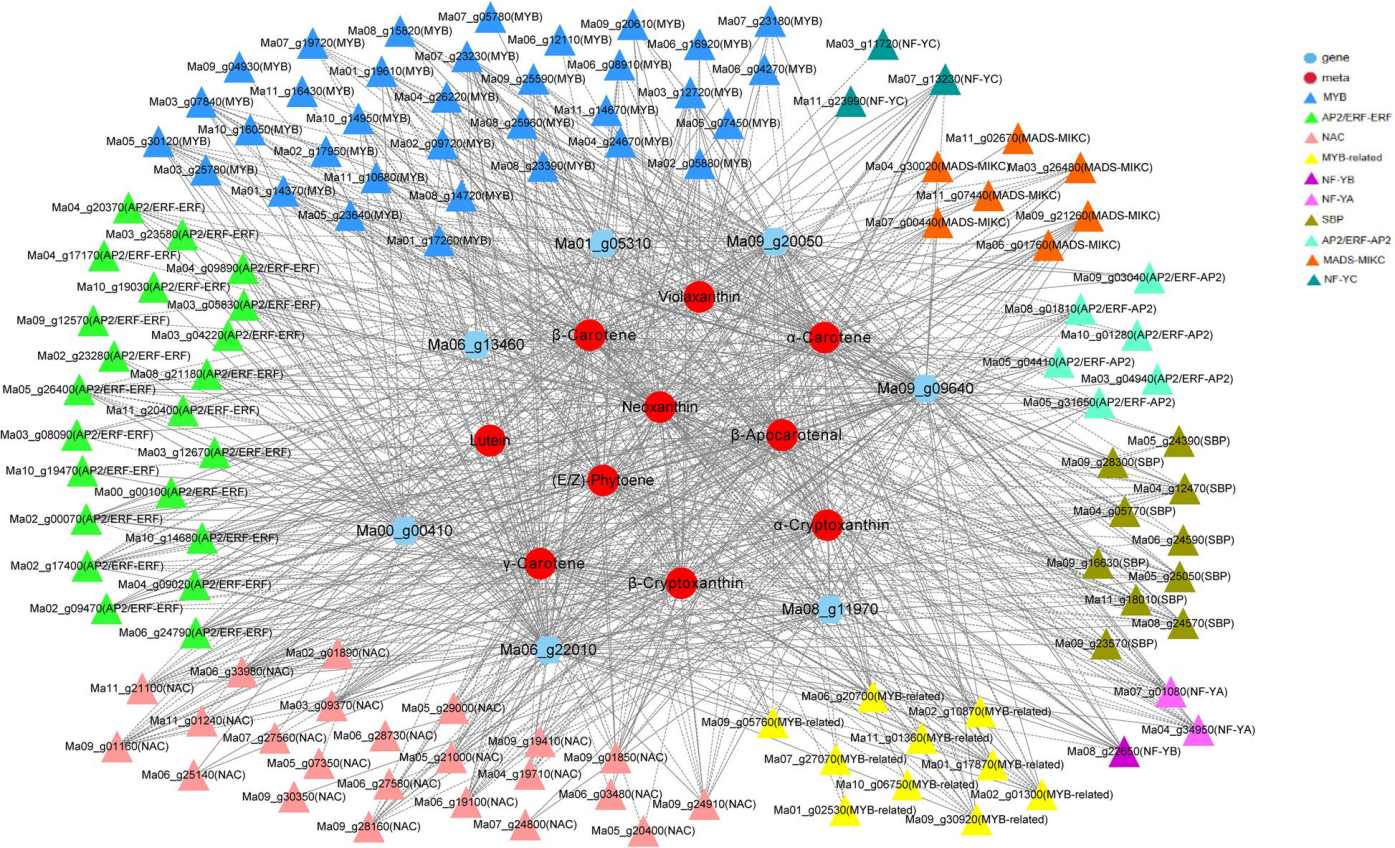
The co-expression network revealed synthetic characteristics and regulators of carotenoid biosynthesis during fruit development. Circular, hexagonal, and triangular nodes represent metabolites, enzyme-coding genes, and transcription factors, respectively. Grey solid lines connected to the nodes depict positive correlations, and dashed lines depict negative interactions

A correlation network was constructed combining 10 metabolites, 7 enzyme genes, and 108 transcription factors related to carotenoid biosynthesis. Only the correlation pairs with a Pearson correlation coefficient > 0.8 were included in this analysis (Fig. 5). The visualized network in Cytoscape showed that a total of 125 nodes were connected, linked by 910 edges. The gene-to-gene FPKM value and gene-to-metabolite accumulation pattern revealed that 351 and 559 pairs of nodes respectively showed positive and negative correlations.

Lycopene β-cyclase (LCYB) is a key enzyme catalyzing the biosynthesis of α-carotene and β-carotene. In Fig. 5, 79 (15 upregulated and 64 downregulated) differentially expressed transcription factor genes were filtered by direct correlation with the gene encoding LCYB.

### Validation of transcriptomic data by quantitative real-time PCR (qRT-PCR)

**Fig. 6:**
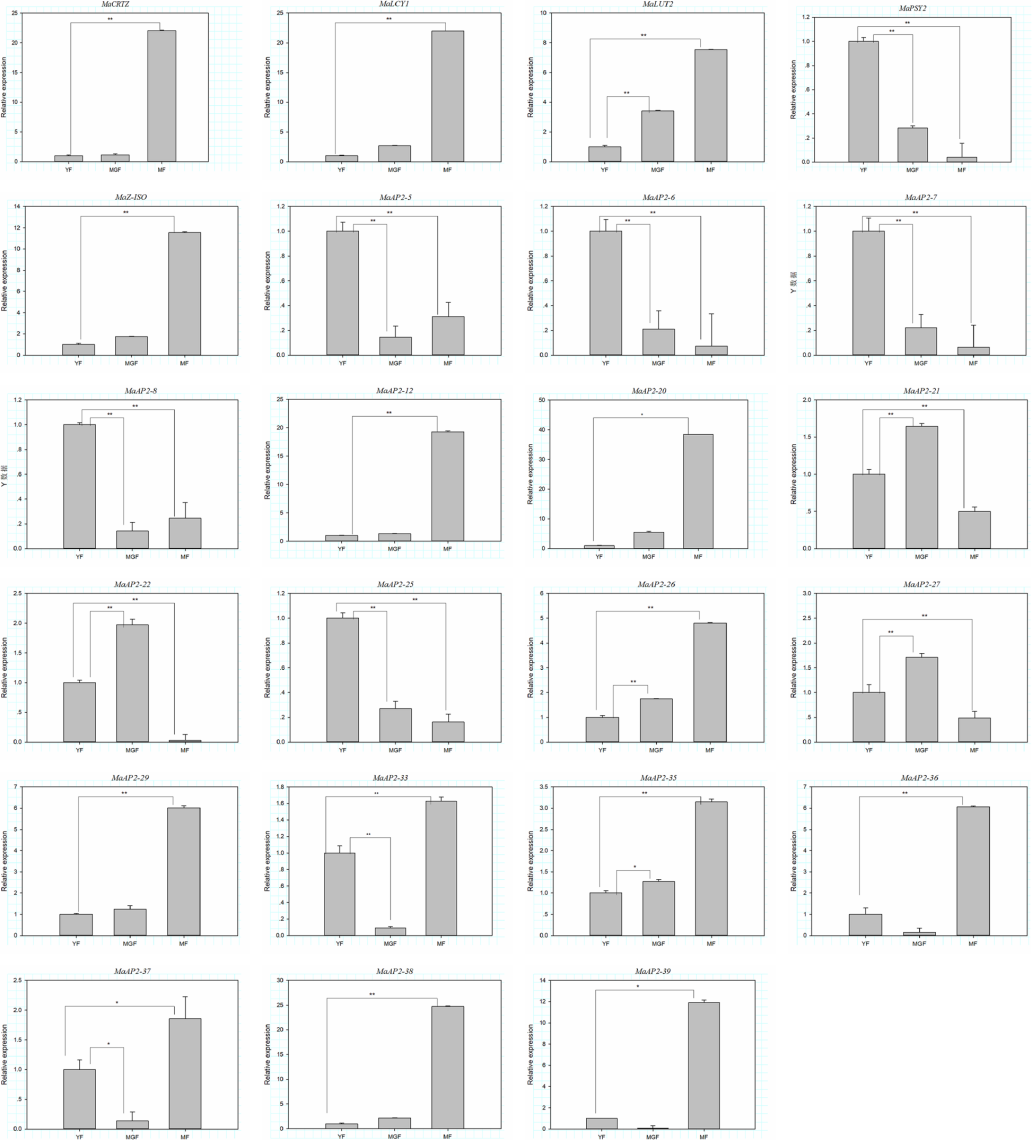
Validation of transcriptomic data by quantitative real-time PCR

A total of 23 DEGs (5 carotenoid biosynthetic pathway genes, 18 transcription factor genes) were used to analyze their expression levels in YF (young fruit), MGF (mature green fruit), and MF (mature fruit) using RT-qPCR to validate the key RNA-Seq results. The expression patterns of these genes were similar to the RNA-Seq results, with correlation coefficients (R^2^) > 0.91 (Fig. 6). The results validated the relevance of the RNA-Seq data, and RT-qPCR showed good consistency for upregulated and downregulated gene expressions.

## Discussion

Carotenoids are widely distributed secondary metabolites that are not only crucial in plant physiology but also beneficial to human health as dietary components [27]. A total of 18 carotenoids were detected by the LC-MS/MS in the present study to investigate the accumulation pattern of carotenoids during the entire developmental period of fruit. However, seven carotenoids remained undetected in this study due to the lower carotenoid content in the sample than the detection limit of the instrument or the absence of carotenoid in the sample. A previous study revealed that α-carotene, β-carotene, and lutein displayed a dramatic increase with banana fruit development [28, 29]. This finding was consistent with the obtained results that most of the carotenoid compounds were undetectable or at considerably low levels at young fruits but markedly increased at the mature green and/or mature fruits. These results all suggest that the synthesis of carotenoids mainly occurs in the middle and late stages of fruit development [28, 29].

RNA sequencing of the samples at three critical developmental stages was performed to understand the genome-wide expression patterns during fruit development. A large number of DEGs across the samples revealed a stage-specific transcriptome profile during fruit development [30]. The GO analysis classified 18,839, 17,800, and 17,469 genes into the biological process, cell component, and molecular function, respectively. These function annotations demonstrated that the gene expressed in banana encodes diverse metabolism-related proteins [23]. KEGG analysis revealed that DEGs were mainly involved in the biosynthesis of secondary metabolites, arachidonic acid metabolism, plant hormone signal transduction, and endocrine and other factor-regulated calcium reabsorption. This study focused on differential carotenoid accumulation during fruit development. The carotenoid accumulation in plants is a complex process associated with the expression of genes involved in carotenoid biosynthesis, degradation, and storage [31]. Carotenoid biosynthesis was enriched in the comparison of young and mature green fruits. Seven DEGs involved in carotenoid biosynthesis were identified, suggesting that these genes may be responsible for the differential carotenoid accumulation during fruit development. A putative road map of carotenoid biosynthesis was also drawn. Notably, most of the DEGs gradually increased with fruit development, which is consistent with the carotenoid metabolic characteristics discussed above and the previous reports [28, 32]. In the current study, the gene encoding Z-ISO gradually increased with fruit development, which is directly correlated with the accumulation of lycopene [28].

The expression of gene encoding lycopene β-cyclase (LCYB), lycopene ε-cyclase (LCYE), and β-carotene hydroxylase gradually increased with fruit development to verify the high contents of carotenoid at the middle and late stages of fruit development. Moreover, the expression level of the gene encoding violaxanthin de-epoxidase (VDE) gradually decreased with fruit development, which resulted in the low content of violaxanthin in mature green and mature fruits. These results suggested that the content of carotenoids is closely related to the expression of structural genes [33].

The transcriptional regulation of carotenoid biosynthetic genes is the first level and an important control mechanism for carotenoid production in fruits [34]. Transcription factors are critical for the regulation of these biosynthetic gene expressions. LCYB is crucial in branching the metabolic flux into either α-carotene in β, ε-branch or β-carotene in β, β-branch of the pathway [34–36]. In the present study, co-expression network analysis revealed that 79 differentially expressed transcription factor genes may be responsible for the regulation of LCYB. The functional analysis of these DEGs will contribute to the understanding regarding the molecular mechanism of carotenoid accumulation in bananas.

## Conclusion

The mechanisms of carotenoid accumulation during banana fruit development were analyzed in this study by using the dynamic metabolites, transcriptome, and qRT-PCR. A total of 11 carotenoid compounds were identified, and most of these compounds had high contents of carotenoid at the middle and late stages of fruit development. Furthermore, a series of carotenoid biosynthetic and regulatory genes were analyzed by RNA-seq and qRT-PCR. Collectively, these findings provide new information on the mechanisms of carotenoid accumulation during banana fruit development and a series of candidate genes with applications in the breeding of special banana subgroups with high carotenoid contents. It is difficult to improve fruit quality by conventional breeding, however molecular breeding which uses gene editing technology might breed directionally high carotenoid content of banana.

## Methods

### Plant materials and treatment

The Xiangfen1 banana plants used in this study were planted in an orchard at South Subtropical Crop Research Institute, Chinese Academy of Tropical Agricultural Science, Zhanjiang, Guangdong, China (21°27 N, 110°35′E). Xiangfen1 banana fruit samples at three different developmental stages (cut off flower days 45, 85, and 85 + 3) were collected from the banana plantation. The fruits collected on the 3 days (days 45, 85, and 85 + 3) represented three typical samples of banana (young, mature green, and mature fruits, respectively). All flesh samples were immediately frozen in liquid nitrogen and stored at −80 °C until further use.

### Chemicals and reagents

Methanol (MeOH), Ethanol (EtOH), Acetone, Methyl tert-butyl ether and BHT were purchased from Merck (Darmstadt, Germany). MilliQ water (Millipore, Bradford, USA) was used in all experiments. All of the standards were purchased from Olchemim Ltd. (Olomouc, Czech Republic) and Sigma (St. Louis, MO, USA). Formic acid was obtained from Sigma. The stock solutions of standards were prepared at the concentration of 1 mg/mL. All stock solutions were stored at -20 °C.

### Sample preparation and extraction

Fresh plant materials were freeze dried, and stored at − 80 °C until needed. All analyses were performed in triplicate. Then dried plant materials were homogenized and powdered in a mill. 50 mg of dried powder was extracted with mixed solution of n-hexane: acetone: ethanol, and add internal standard. The extract was vortexed for 20 min at room temperature. The supernatants were collected after centrifugation. The residue was re-extracted and repeat the steps above. Both supernatants were collected and then evaporated to dryness under nitrogen gas stream, reconstituted in mixed solution of methanol: MTBE. The solution was filtered through 0.22 μm filter for further LC-MS analysis [37].

### HPLC conditions

The sample extracts were analyzed using an LC- APCI-MS/MS system (UHPLC, ExionLC AD, https://sciex.com.cn/; MS, Applied Biosystems 6500 Triple Quadrupole, https://sciex.com.cn/). The analytical conditions were as follow, HPLC: column, YMC C30 (3 μm, 2 mm*100 mm); solvent system, methanol: acetonitrile (3:1,V/V) (0.01% BHT, 0.1% formic acid): methyl tert-butyl ether (0.01% BHT); gradient program, 100:0 V/V at 0 min, 100:0 V/V at 3 min, 58:42 V/V at 6 min, 20:80 V/V at 8 min, 5:95 V/V at 9 min,100:0 V/V at 9.1 min,100:0 V/V at 11 min; flow rate, 0.8 mL/min; temperature, 28 °C; injection volume: 2 μL [38].

### APCI-q trap-MS/MS

API 6500 Q TRAP LC/MS/MS System, equipped with an APCI Turbo Ion-Spray interface, operating in a positive ion mode and controlled by Analyst 1.6.3 software (AB Sciex). The APCI source operation parameters were as follow: ion source, APCI+; source temperature 350 °C; curtain gas (CUR) were set at 25.0 psi; the collision gas (CAD) was medium. DP and CE for individual MRM transition was done with further DP and CE optimization. A specific set of MRM transitions were monitored for each period according to the carotenoids eluted within this period [39].

### Detection of carotenoids

α-Carotene, β-Carotene, γ-Carotene, ε-Carotene, Lutein, Violaxanthin, Antheraxanthin, Neoxanthin, Zeaxanthin, β-Cryptoxanthin, α-Cryptoxanthin, all-trans-Lycopene, Phytofluene, (E/Z)-Phytoene, Astaxanthin, Capsanthin, Apocarotenal and Capsorubin contents were detected by MetWare (http://www.metware.cn/) based on the AB Sciex QTRAP6500 LC-MS/MS platform.

### RT-qPCR validation

RT-qPCR was applied to investigate gene expression patterns. First-strand cDNA was generated from 1 μg total RNA isolated from the seven pericarp samples using the PrimeScript™ RT reagent kit (TaKaRa, Japan). RT-qPCR primers were designed using Primer Premier 5.0 software (Premier, Canada) and synthesized by Sangon Biotech (Shanghai, China) Co., Ltd. The relative expression level of the genes were calculated using Eq. 2^−ΔΔ*C* t^.

### Statistical analysis

To reduce the dimension of data and simplify transcriptome data, principal component analysis (PCA), a multivariate statistical analysis method, was used in this study. The differential metabolites and genes were annotated using the Kyoto Encyclopedia of Genes and Genomes (KEGG) Pathway database (http://www.kegg.jp/kegg/pathway.html).

## Data Availability

The datasets generated during the current study are available from the corresponding author on reasonable request. All raw read data were deposited in the Sequence Read Archive (SRA) in NCBI with the Bioproject ID: PRJNA776816 (https://dataview.ncbi.nlm.nih.gov/object/PRJNA776816?reviewer=ba2olltnfd86gmonebc83286av).

## Abbreviations

HPLC: High-performance liquid chromatography
UHPLC: Ultra high-performance liquid chromatography
LC-MS: Liquid chromatography-tandem mass spectrometry
IPP: Isopentenyl diphosphate
DMAPP: Dimethylallyl diphosphate
GGPP: Geranylgeranyl pyrophosphates
PCA: Principal component analysis
